# Supplementary material for: Cyclic Peptide Mimotopes for the Detection of Serum Anti–ATIC Autoantibody Biomarker in Hepato-Cellular Carcinoma
Source: Int J Mol Sci. 2020 Dec 19;21(24):9718. doi: 10.3390/ijms21249718 (PMC7766137; doi:10.3390/ijms21249718)
Supplement: Supplementary file 1 [file ijms-21-09718-s001.pdf]

Supplementary data Fig. S1

HiTrap-Q ion exchange chromatography
- LNCap/LN3 cell lysate (RIPA buffer, 6.8mg)
- Buffer A : PBS
- Buffer B : PBS, 1M NaCl

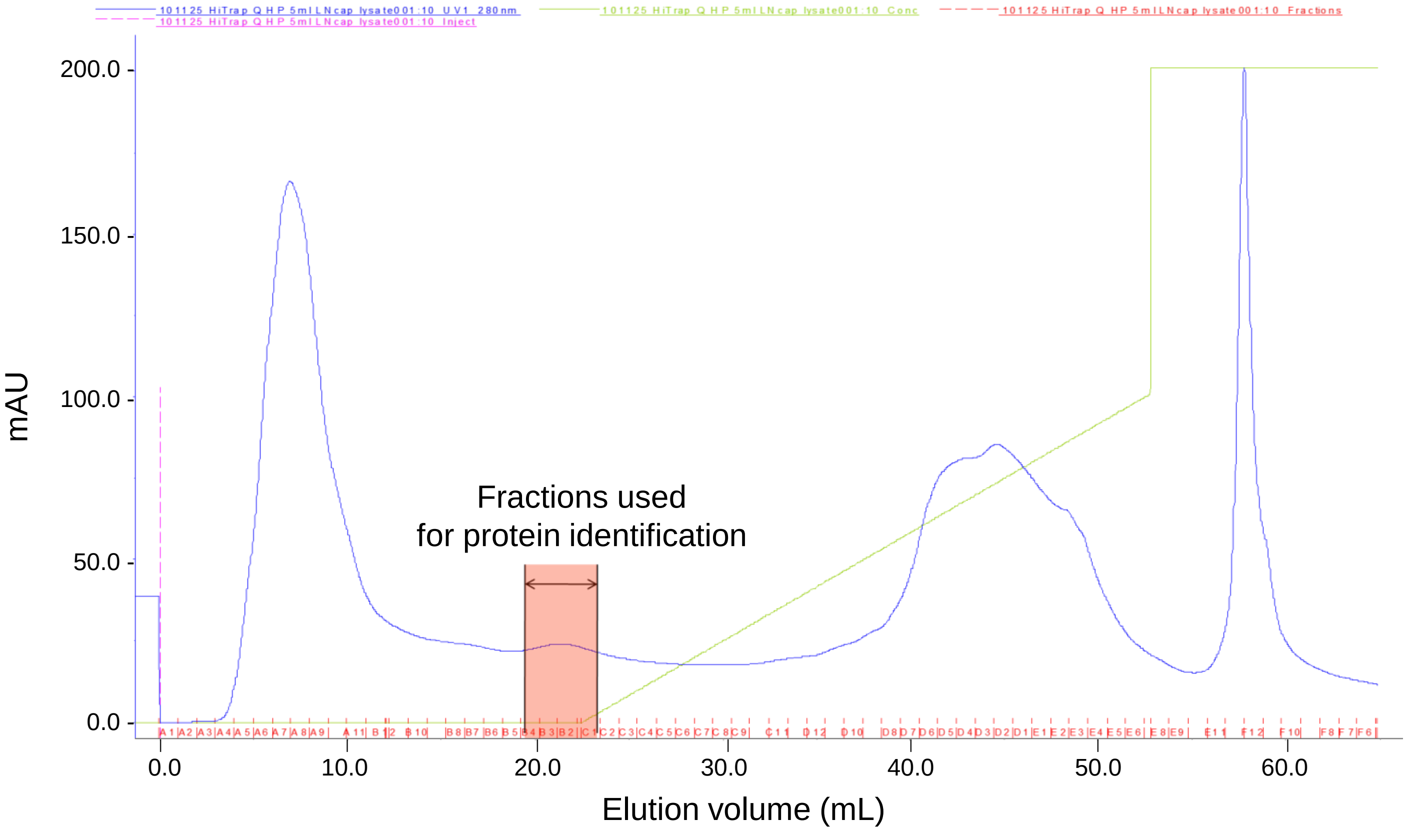

Supplementary Figure S1. Enrichment of XC154 antigen by fractionation of LNCap/LN3 cell lysate by anion exchange chromatography. Cell lysates with RIPA buffer were fractionated using Hitrap Q anion-exchange chromatography column by salt-gradient elution. The fractions enriched with XC154 antigen (B4~C1), which was confirmed by Western blotting analysis, were pooled and its concentrate was subjected to preparative 10% SDS-PAGE. The protein band corresponding to the XC154 antigen was excised and in-gel digested with trypsin. The peptides were extracted from gel matrix and analyzed by LC/ESI-MS/MS. The identified protein candidates are listed in Table 1.

Supplementary data_Fig. S2

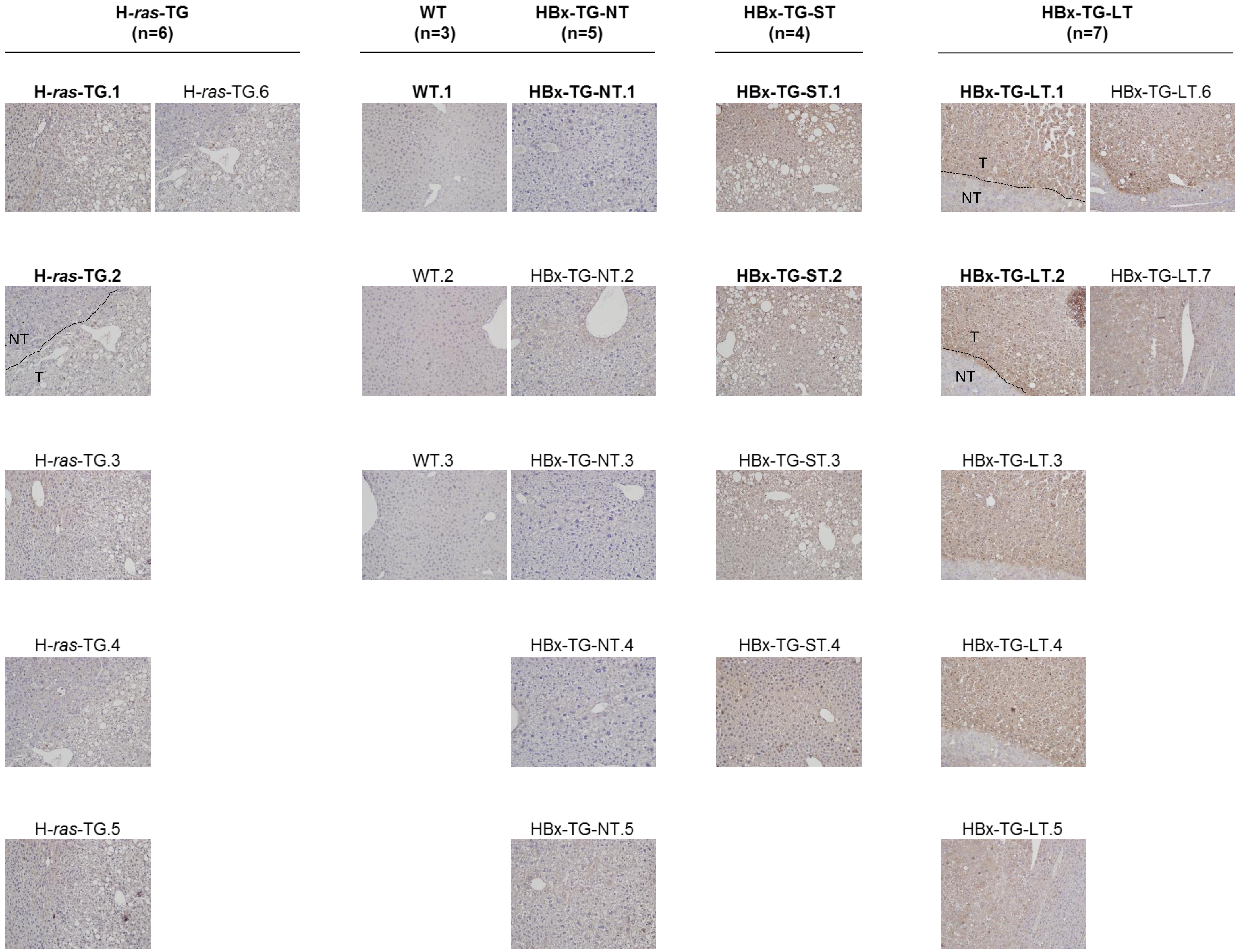

Supplementary Figure S2. Immunohistochemical analysis of ATIC in liver tissues of HCC model mice. Liver tissues from wild type control mice (Non-Tg: WT) were also stained. NT: non-tumor, T: tumor region, H-ras12V-Tg (n=6), Non-Tg (n=3), HBX-Tg-nonT: HBX-transgenic mouse without tumor (n=5), HBX-Tg-ST: HBX-transgenic mouse with small tumor (n=6), HBX-Tg-LT: HBX-transgenic mouse with large tumor (n=7). Representative images (names in bold fonts) were shown in Fig. 2.

Supplementary data_Fig. S3

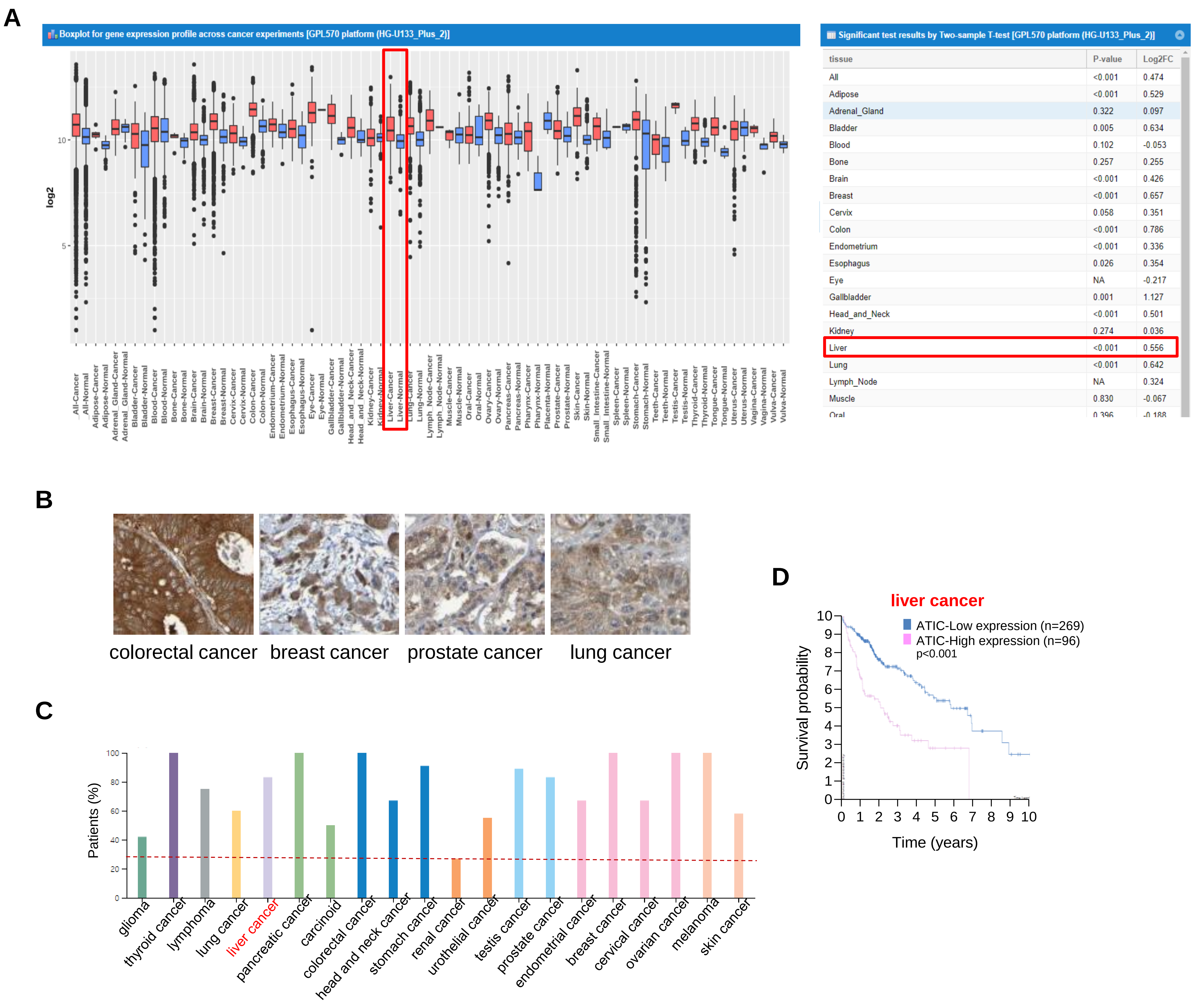

tissue	P-value	Log2FC
All	<0.001	0.474
Adipose	<0.001	0.529
Adrenal_Gland	0.322	0.097
Bladder	0.005	0.634
Blood	0.102	-0.053
Bone	0.257	0.255
Brain	<0.001	0.426
Breast	<0.001	0.657
Cervix	0.058	0.351
Colon	<0.001	0.786
Endometrium	<0.001	0.336
Esophagus	0.026	0.354
Eye	NA	-0.217
Gallbladder	0.001	1.127
Head_and_Neck	<0.001	0.501
Kidney	0.274	0.036
Liver	<0.001	0.556
Lung	<0.001	0.642
Lymph_Node	NA	0.324
Muscle	0.830	-0.067
Oral	0.396	-0.188

Supplementary Figure S3. The expression analysis of ATIC. (**A**) The expression analysis of *ATIC* by GENT2 (http://gent2.appex.kr/gent2/) showed a significant increase of *ATIC* expression in various human cancers, including breast, colon, lung, and liver cancers. (**B, C**) Immunohistochemical analysis in the TCGA database (https://www.proteinatlas.org/ENSG00000138363-ATIC/pathology) also showed the elevation of ATIC protein in various tumors. (**D**) Kaplan-Meier survival analysis (https://www.proteinatlas.org/ENSG00000138363-ATIC/pathology) shows that ATIC is a poor prognosis marker of liver cancer.

Supplementary data Fig. S4

A. Protein BLAST of Bifunctional purine biosynthesis protein ATIC

Species	Sequence ID	Length (a.a.)	Score	Identities	Positives	Gaps
**[Mus musculus]**	**NP_080471.2**	**592**	**1219 bits(3155)**	**592/592(100%)**	**592/592(100%)**	**0/592(0%)**
[Mus musculus]	BAB27060.1	592	1216 bits(3147)	591/592(99%)	591/592(99%)	0/592(0%)
[Mus caroli]	XP_021013660.1	592	1215 bits(3143)	589/592(99%)	591/592(99%)	0/592(0%)
[Mus pahari]	XP_021053512.1L	592	1205 bits(3117)	582/592(98%)	588/592(99%)	0/592(0%)
[Rattus norvegicus]	NP_112276.2	592	1197 bits(3097)	577/592(97%)	587/592(99%)	0/592(0%)
[Rattus norvegicus]	BAA22837.1	592	1194 bits(3088)	576/592(97%)	586/592(98%)	0/592(0%).
[Peromyscus manicula tus bairdii]	XP_006972304.1	592	1184 bits(3062)	569/592(96%)	579/592(97%)	0/592(0%)
**[Homo sapiens]**	**NP_004035.2**	**592**	**1134 bits(2932)**	**539/592(91%)**	**567/592(95%)**	**0/592(0%)**

B. Kolaskar & Tongaonkar Antigenicity Analysis

Human ATIC

Center position: 4 Window size: 7 Threshold: 1.040 Recalculate

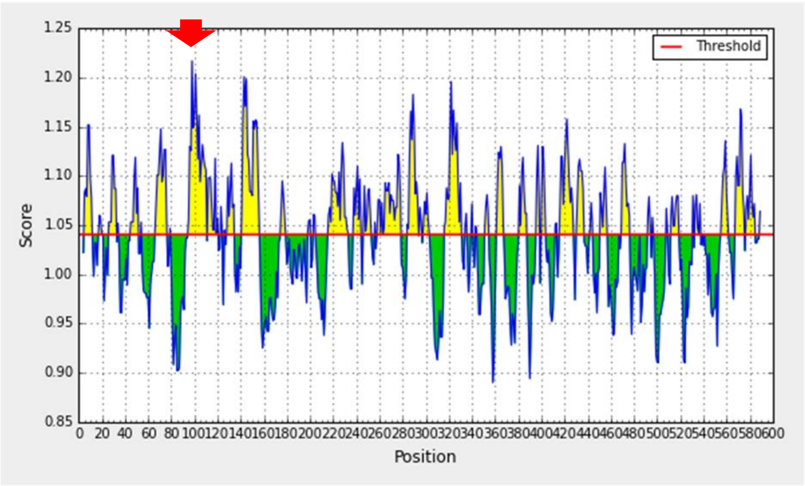

Average: 1.040 Minimum: 0.890 Maximum: 1.217

Mouse ATIC

Center position: 4 Window size: 7 Threshold: 1.044 Recalculate

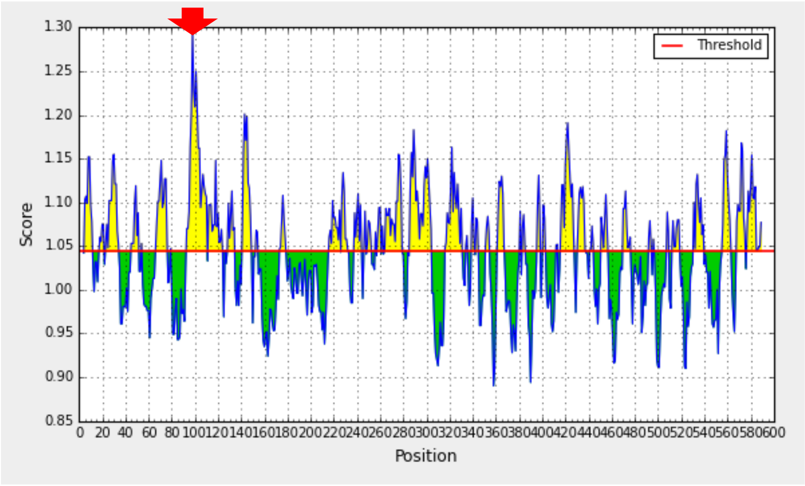

Average: 1.044 Minimum: 0.890 Maximum: 1.295

Antigenic sequences analyzed by Kolaskar & Tongaonkar Antigenicity method

```
hATIC    1  MAPGQLALFSVSDKTGLVEFARNLTALGLNLVASGGTAKALRDAGLAVRD  50
            |||*||||||||||||||||||*|**|||*||||||||||*|||||||||
mATIC    1  MAPSQLALFSVSDKTGLVEFARSLASLGLSLVASGGTAKAIRDAGLAVRD  50

hATIC   51  VSELTGFPEMLGGRVKTLHPAVHAGILARNIPEDNADMARLDFNLIRVVA  100
            ||||||||||||||||||||||||||||||||||*|||||||||||*|||*
mATIC   51  VSELTGFPEMLGGRVKTLHPAVHAGILARNIPEDAADMARLDFNLVRVVV  100

hATIC  101  CNLYPFVKTVASPGVTVEEAVEQIDIGGVTLLRAAAKNHARVTVVCEPED  150
            |||||||||||||*||||*|||||||||||||||||||||||||||||||
mATIC  101  CNLYPFVKTVASPDVTVEAAVEQIDIGGVTLLRAAAKNHARVTVVCEPED  150

hATIC  151  YVVVSTEMQSSESKDTSLETRRQLALKAFTHTAQYDEAISDYFRKQYSKG  200
            |**|**||*|**||||||||||*|||||||||||||||||||||||||||
mATIC  151  YAGVAAEMHGSDSKDTSLETRRHLALKAFTHTAQYDEAISDYFRKQYSKG  200

hATIC  201  VSQMPLRYGMNPHQTPAQLYTLQPKLPITVLNGAPGFINLCDALNAWQLV  250
            *||||||||||||||||||||||||*||||||||||||||||||||||||
mATIC  201  ISQMPLRYGMNPHQTPAQLYTLKPKLPITVLNGAPGFINLCDALNAWQLV  250

hATIC  251  KELKEALGIPAAASFKHVSPAGAAVGIPLSEDEAKVCMVYDLYKTLTPIS  300
            *||**|**||||||||||||||||||*|||||||*||||||||*|||||*
mATIC  251  TELRGAVDIPAAASFKHVSPAGAAVGVPLSEDEARVCMVYDLYPTLTPLA  300

hATIC  301  AAYARARGADRMSSFGDFVALSDVCDVPTAKIISREVSDGIIAPGYEEEA  351
            *||||||||||||||||||||||*|||||||||||||||||*||||||||
mATIC  301  VAYARARGADRMSSFGDFVALSDICDVPTAKIISREVSDGIVAPGYEEEA  351

hATIC  351  LTILSKKKNGNYCVLQMDQSYKPDENEVRTLFGLHLSQKRNNGVVDKSLF  400
            |*||||||||||||||||||||||||||||||||*|||||||||||||||
mATIC  351  LKILSKKKNGNYCVLQMDQSYKPDENEVRTLFGLRLSQKRNNGVVDKSLF  400

hATIC  401  SNVVTKNKDLPESALRDLIVATIAVKYTQSNSVCYAKNGQVIGIGAGQQS  451
            ||*||||||||||||||||||||||||||||||||||*||||||||||||
mATIC  401  SNIVTKNKDLPESALRDLIVATVAVKYTQSNSVCYAKDGQVIGIGAGQQS  451

hATIC  451  RIHCTRLAGDKANYWWLRHHPQVLSMKFKTGVKRAEISNAIDQYVTGTIG  500
            |||||||||||||*|||||||||||||||*||||||||||||||||||||
mATIC  451  RIHCTRLAGDKANSWWLRHHPRVLSMKFKAGVKRAEISNAIDQYVTGTIG  500

hATIC  501  EDEDLIKWKALFEEVPELLTEAEKKEWVEKLTEVSISSDAFFPFRDNVDR  551
            |||||*||*|||||||||||||||||||*||**||*||||||||||||||
mATIC  501  EGEDLVKWEALFEEVPELLTEAEKKEWVDKLSGVSVSSDAFFPFRDNVDR  551

hATIC  551  AKRSGVAYIAAPSGSAADKVVIEACDELGIILAHTNLRLFHH  592
            |||||||||*|||||*||||||||||||||*||||*||||||
mATIC  551  AKRSGVAYIVAPSGSTADKVVIEACDELGIVLAHTDLRLFHH  592
```

Supplementary data_Fig. S5

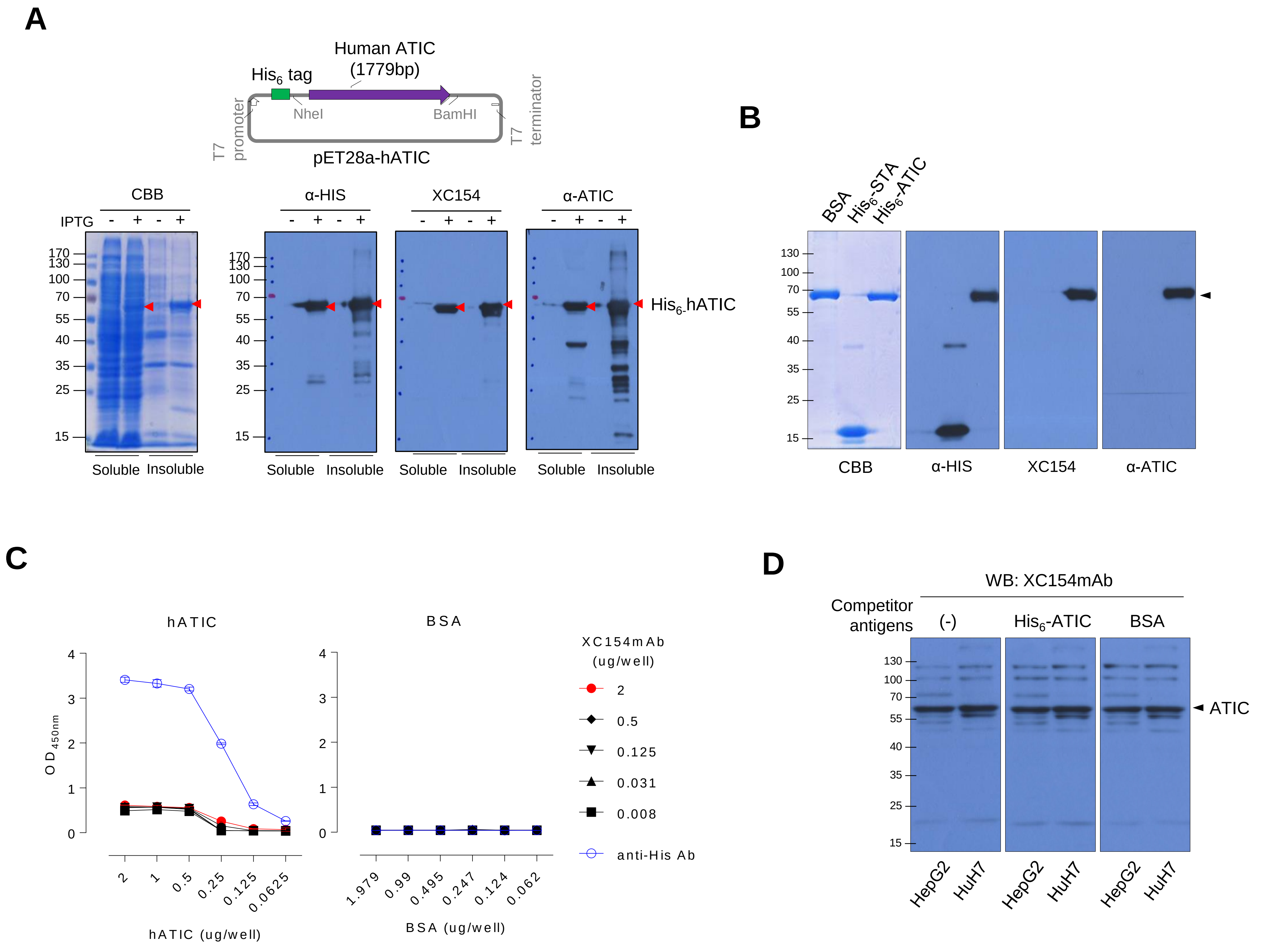

Supplementary Figure S5. The expression of human ATIC and the binding to XC154mAb. (**A**) The expression of recombinant human ATIC (hATIC) in T7 shuffle *E. coli* cells. The human ATIC gene was amplified from cDNA of HepG2 cells and cloned into the histidine (His_6)-tagged pET28a expression vector. The expression vector was transformed in T7 shuffle *E. coli* cells, and the protein expression was induced with IPTG (final 1 mM). The cell lysates were prepared by sonication, and the soluble and insoluble fractions were separated by centrifugation. The cell lysates (40 μg/lane) were resolved on 10% SDS-PAGE and detected using western blot analysis with anti-His Ab, anti-ATIC antibody, and XC154 mAb. (**B**) His_6-tagged hATIC was purified by using Talon metal-affinity resin and detected by SDS-PAGE and western blot analysis. (**C**) Recombinant hATIC was detected with XC154 mAb by ELISA. The coating antigen was also estimated by ELISA with anti-His Ab. ELISA with BSA was performed as a control. (**D**) Competitive western blot analysis of ATIC with recombinant hATIC. Cell lysates (40 μg/lane) were separated on 10% SDS-PAGE and treated with XC154 mAb, pre-incubated with competitors (5 μg Ab with 7 μg competitor).

Supplementary data_Fig. S6

Cohort 1

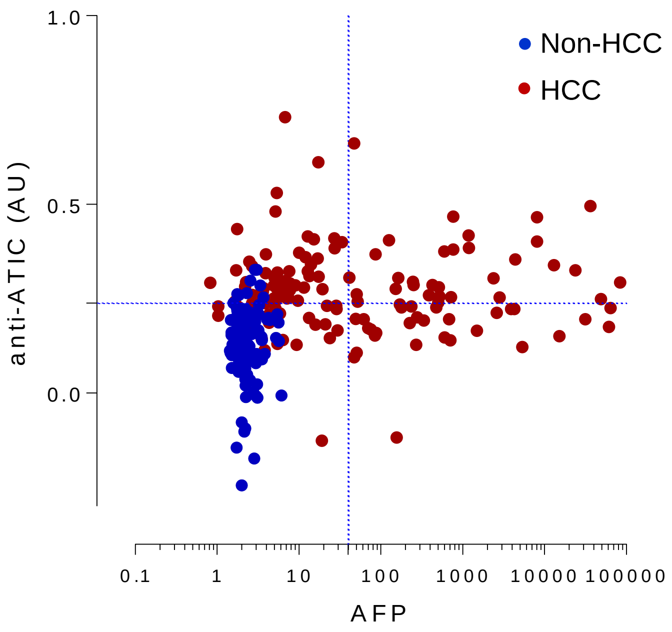

Cohort 2

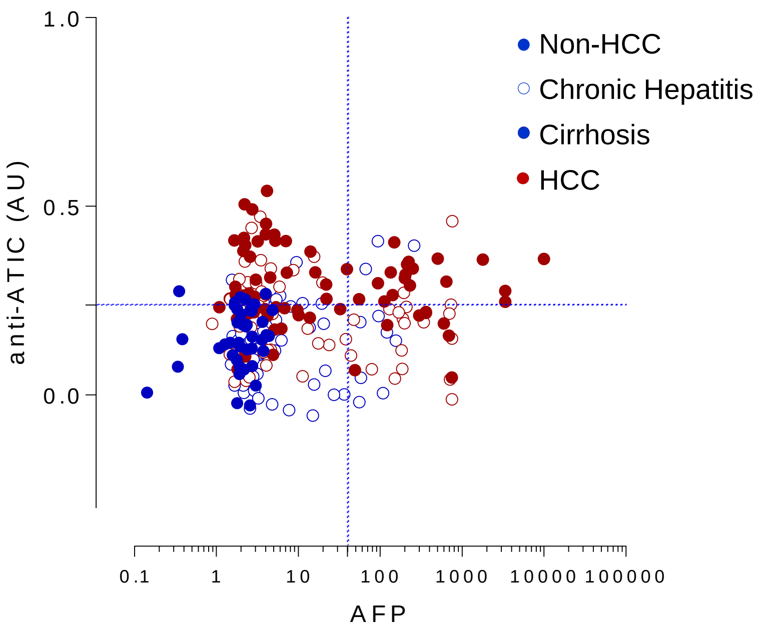

	AFP vs. anti-ATIC
Pearson r	
r	0.08659
95% confidence interval	-0.03497 to 0.2056
P (two-tailed)	0.1623
P value summary	ns
Significant? (alpha = 0.05)	No
Number of XY Pairs	262
Number of non-HCC	118
Number of HCC	144

	AFP vs. anti-ATIC
Pearson r	
r	0.1072
95% confidence interval	-0.01997 to 0.2310
P (two-tailed)	0.0983
P value summary	ns
Significant? (alpha = 0.05)	No
Number of XY Pairs	245
Number of Non-HCC	44
Number of Chronic Hepatitis	64
Number of Cirrhosis	64
Number of HCC	73

Supplementary data_Fig. S7

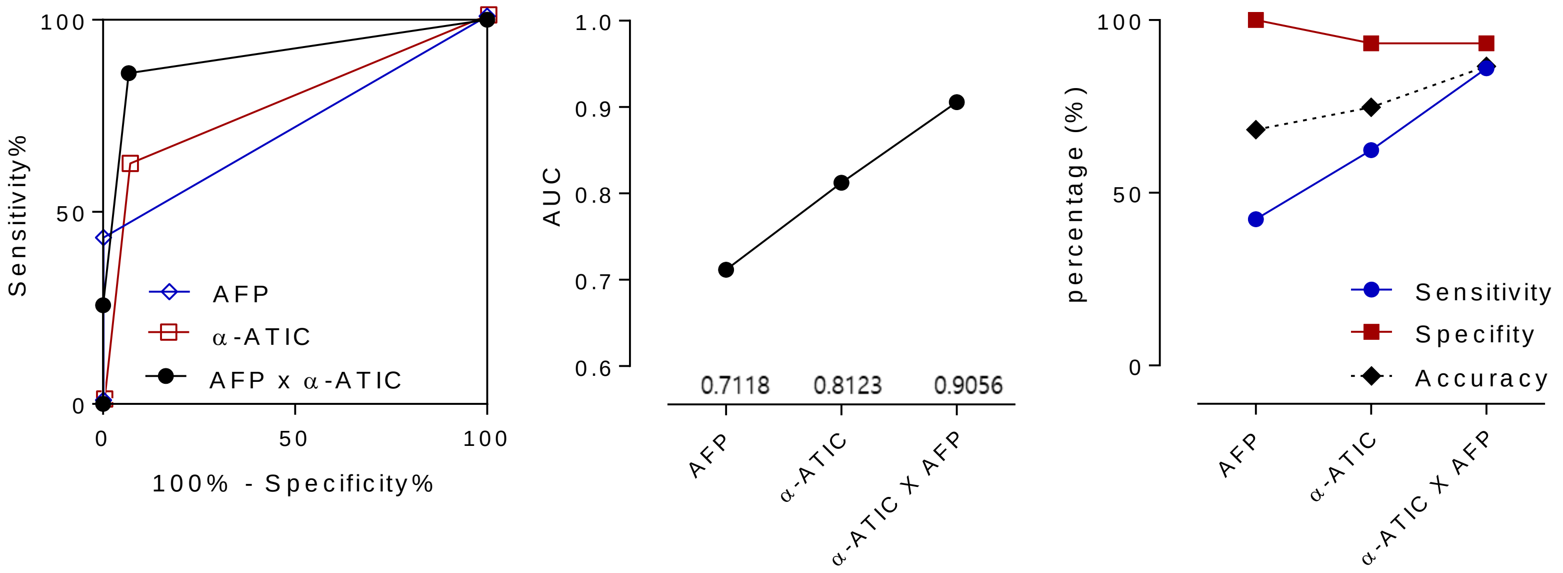

	AFP	α-ATIC	AFP x α-ATIC
Area under the ROC curve			
Area	0.7118	0.7733	0.9056
Std. Error	0.03153	0.02922	0.01983
95% confidence interval	0.65 to 0.7736	0.716 to 0.8305	0.8667 to 0.9445
P value	<0.0001	<0.0001	<0.0001
Data			
Controls (Normal)	118	118	118
Patients (HCC)	144	144	144
Missing Controls	0	0	0
Missing Patients	0	0	0
